# Dynamic Trends in Surgical Oromaxillofacial Trauma Epidemiology: A Comparative Study of Pre-COVID-19 and COVID-19 Periods in Tertiary Referral Hospitals in Madrid

**DOI:** 10.3390/jcm13071947

**Published:** 2024-03-27

**Authors:** Angela Sada-Urmeneta, Manuel Tousidonis, Carlos Navarro-Cuellar, Santiago Ochandiano, Ignacio Navarro-Cuellar, Saad Khayat, Gonzalo Ruiz-de-León, Marta Benito-Anguita, Sara Alvarez-Mokthari, Eduardo Olavarria, Gregorio Sanchez-Aniceto, Sonia Herrero-Alvarez, Oscar de la Sen-Corcuera, Anna-Maria Simon-Flores, Fernando Almeida-Parra, Iñigo Aragon-Niño, Jose-Luis del-Castillo, Jose-Ignacio Salmeron

**Affiliations:** 1 Department of Oral and Maxillofacial Surgery, Hospital General Universitario Gregorio Marañón, 28009 Madrid, Spainsochandiano@hotmail.com (S.O.); mbanguita@salud.madrid.org (M.B.-A.);; 2 Department of Oral and Maxillofacial Surgery, Hospital Universitario Doce de Octubre, 28041 Madrid, Spain; 3 Department of Oral and Maxillofacial Surgery, Hospital Universitario Clínico San Carlos, 28040 Madrid, Spainodelasen@gmail.com (O.d.l.S.-C.); 4 Department of Oral and Maxillofacial Surgery, Hospital Universitario Ramón y Cajal, 28034 Madrid, Spain; 5 Department of Oral and Maxillofacial Surgery, Hospital Universitario La Paz, 28046 Madrid, Spain; inigo.aragon@salud.madrid.org (I.A.-N.);

**Keywords:** SARS-CoV-2, COVID-19, orofacial trauma, maxillofacial trauma, epidemiology, public health

## Abstract

**Introduction**: The COVID-19 pandemic has induced profound societal and healthcare transformations globally. **Material and methods**: This multicenter retrospective study aimed to assess potential shifts in the epidemiology and management of oromaxillofacial trauma requiring surgical intervention over a 1-year period encompassing the onset of the COVID-19 pandemic, in comparison to the preceding year. The parameters investigated included age, sex, injury mechanisms, fractured bones, and treatment modalities. The statistical significance was set at *p* < 0.05. **Results**: A notable 39.36% reduction in oromaxillofacial fractures was identified (*p* < 0.001), with no significant alterations in sex distribution, types of fractured bones, or treatment modalities. An appreciable increase in mean age was observed (35.92 vs. 40.26) (*p* = 0.006). Analysis of the causes of oromaxillofacial trauma revealed diminished incidents of interpersonal violence (41% vs. 35%) and sports-related injuries (14% vs. 8%), alongside an escalation in cases attributed to falls (27% vs. 35%), precipitation events (2% vs. 5%), and traffic accidents (12% vs. 13%). The mandible emerged as the most frequently fractured bone. **Conclusion**: In conclusion, the COVID-19 pandemic has decreased the number of maxillofacial fractures treated surgically and has changed the epidemiology and the etiology of facial traumas.

## 1. Introduction

The World Health Organization officially declared COVID-19 a pandemic on 11 March 2020 [1,2,3]. By November 2021, the worldwide tally surpassed 257.4 million confirmed COVID-19 cases, with 5.15 million associated fatalities. In response to the pandemic classification in March 2020, nations worldwide implemented diverse social distancing measures. This led to an immediate paradigm shift in the operational strategies of healthcare institutions, including maxillofacial surgery departments. Scheduled activities were suspended to prioritize the management of non-deferrable acute illnesses and trauma emergencies [4].

In Europe, during the first and most lethal wave, Spain was one of the most affected countries, challenging health systems and policy makers [4]. To mitigate the spread, the Spanish government implemented a stringent lockdown on 14 March 2020, lasting 50 days. The lockdown measures included restrictions on movements, activities, sports, and travel; prohibitions on social outings and gatherings; prolonged periods of confinement; and the mandatory adoption of social distancing and facial masks. Subsequently, these measures were gradually eased through three distinct phases, facilitating the gradual restoration of interpersonal relationships [4]. Madrid was the most affected city in Spain, and required the redistribution of sanitary resources to treat the large number of patients admitted with COVID-19, using operating theatres as intensive care units, canceling surgeries, and requiring field hospitals.

The spectrum of oromaxillofacial trauma includes soft tissue and bone, and ranges from the simple to the complex. The epidemiology varies according to local and global demographic factors and reflects a complex interplay of influences, including those related to the environment, economics, age, sex, and mechanism of injury. Despite various studies that have explored the changes in the epidemiology of oromaxillofacial fractures, the causes of these epidemiological changes are variable among the different regions and cultures analyzed [1,5]. An accurate understanding of oromaxillofacial trauma incidence is further complicated by under-reporting and the often-overlooked treatment of minor injuries. 

Surgical oromaxillofacial trauma is defined as a trauma produced in the oromaxillofacial territory that requires treatment in the operating room for the reduction (open or closed) of a fracture of one or more facial bone and/or dentoalveolar fracture. The main etiologies of oromaxillofacial trauma include interpersonal violence, sports-related injuries, accidental falls, and traffic accidents [5].

The COVID-19 pandemic has produced changes in social interactions, a decrease in the number of trips, limitation of sports and outdoor activities, and the implementation of strict lockdowns in various countries for various periods of time [1,2,3,4,5]. These restrictive measures have produced a decrease in the number of trips with cars and motorcycles, sports activities, and social gatherings, described as the main mechanisms of orofacial fractures in the scientific literature. Such changes could potentially alter the epidemiology and etiology of oromaxillofacial trauma, necessitating adjustments to address population needs, identify areas for improvement, and offer recommendations for resource planning and a more efficient surgical practice strategy, particularly in anticipation of future viral outbreaks and population lockdowns. Most studies on oromaxillofacial trauma epidemiology have been conducted at single centers and have often lacked comparisons of etiology and surgical treatment variability with previous years, hindering the assessment of trend changes attributed to COVID-19 [1,5].

This study aimed to evaluate the impact of the COVID-19 pandemic on oromaxillofacial surgical trauma in Madrid. The comparison involved surgical cases treated during a one-year period from the onset of strict lockdown with the corresponding period in the previous year. Reviewing scientific publications to date, only one Spanish study was published that analyzed the epidemiology of maxillofacial trauma during the COVID-19 pandemic [4]. This published study was monocentric and analyzed cases in a region with a lower incidence of COVID-19 than Madrid. Our study is the first multicenter study carried out in Spain to analyze the epidemiology and treatment of maxillofacial trauma, in the area with the highest incidence of COVID-19 in the country, Madrid. The objective was to determine the interannual variation in the incidence, demographic patterns, and characteristics of maxillofacial fractures to identify areas for improvement and provide recommendations on the more efficient planning of resources and a surgical practice strategy that may be useful in the event of new virus outbreaks and further population lockdowns.

## 2. Materials and Methods

A retrospective multicenter comparative cohort study was undertaken, inviting all six tertiary hospitals with oral and maxillofacial surgery units in Madrid: Hospital General Universitario Gregorio Marañón, Hospital General Universitario La Paz, Hospital Universitario 12 Octubre, Hospital Clínico San Carlos, and Hospital Universitario La Princesa. Hospital Universitario La Princesa declined participation.

This study comprised a retrospective multicenter comparative cohort investigation of patients undergoing surgery for oromaxillofacial fractures at oromaxillofacial surgery departments in Madrid (Spain) during both the pre-COVID-19 and COVID-19 periods. The incidence and patient characteristics during the initial year of the coronavirus pandemic were compared with those of a control group from the corresponding period in the previous year. The COVID-19 period was defined as 1 year after the start of strict confinement in Spain in March 2020, with equivalent data collected from patients who underwent surgery in the same period the preceding year, from March 2019 to February 2020 (pre-COVID-19 group). This one-year limit was chosen due to the occurrence of severe government restrictions and subsequent COVID-19 waves during this period, significantly impacting the social life of the Spanish population. Accordingly, two groups were established: (1) pandemic group (PG) or experimental group: between 1 March 2020 and 28 February 2021; and (2) pre-pandemic group (PPG) or control group: between 1 March 2019 and 28 February 2020.

Patients were identified based on the rates of surgical procedures provided by the medical information network of each hospital center. Cases selected through medical record analysis had at least one radiologically proven fracture of a facial bone with surgical treatment indication. Data were collected from all consecutive patients who underwent surgery for maxillofacial fractures from 1 March 2019 to 28 February 2021 across the five participating hospitals.

Inclusion criteria were (1) patients operated under general anesthesia for one or more oromaxillofacial fractures and (2) complete clinical data recorded in their medical histories. Exclusion criteria were (1) patients with oromaxillofacial trauma treated on an outpatient basis or under local anesthesia/sedation; (2) isolated nasal bone fractures without surgical reduction (3) isolated dental fractures; and (4) interventions for post-traumatic complications, sequelae of previous trauma, or removal of osteosynthesis plates. This study adhered to the Declaration of Helsinki regarding medical protocol and ethics, with all patients providing informed consent before surgery. 

Causal mechanisms were categorized as assault, casual fall, traffic (inclusive of road traffic, car accidents, motorbikes, bicycles, and electric scooters), sports, precipitation, and others. Affected bones were further subdivided into the mandible, orbit, orbitomalar and malar, zygomatic arch, naso-orbito-ethmoidal, LeFort (all three types), nasal, dentoalveolar, panfacial, and others. Treatment modalities encompassed open reduction and internal fixation (ORIF), intermaxillary block (IMB), ORIF plus IMB, reduction with Ginestet hook or Gillie’s technique, closed reduction, rhinoseptoplasty, dental splinting, and others.

Data were tabulated in a Microsoft Excel spreadsheet (Microsoft Corporation, Redmond, WA, USA) without recognizing the patient’s identity, which was only accessible to the main researcher. Both patient autonomy and confidentiality were strictly maintained throughout this study. A comparison between groups was conducted to assess the association between monthly incidence and confinement periods. Outcome variables were analyzed using the SPSS statistical program, version 25 (IBM Corporation, Armonk, NY, USA). Continuous variables are expressed as mean with standard deviation and quantitative data as frequencies. Statistical inferences and corresponding figures were generated using the chi-square test and the independent samples *t*-test. The null hypothesis posited that the pandemic did not impact the epidemiology of oromaxillofacial trauma, with *p*-values less than 0.05 considered statistically significant.

## 3. Results

**Figure 1:**
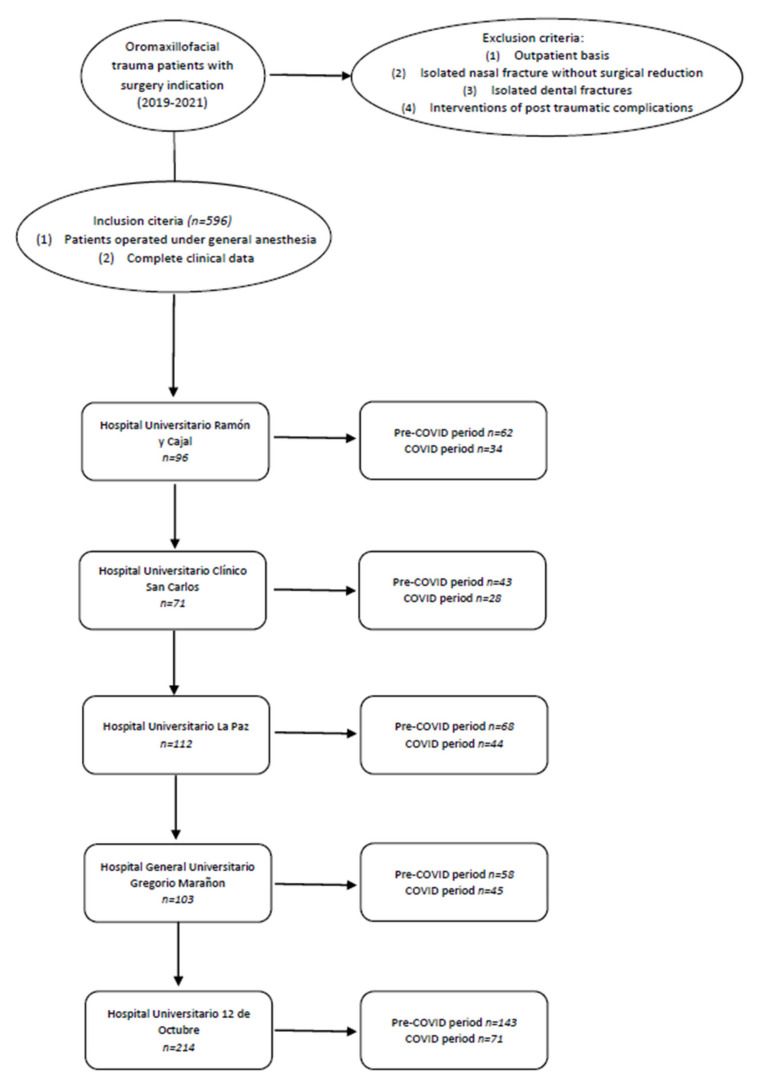
Flowchart that includes the inclusion and exclusion criteria and the number of patients selected by each hospital for both study periods.

A total of 596 patient medical records were analyzed (Figure 1). Patients’ characteristics are summarized in Table 1. 

Of the total of 596 patients analyzed, 371 patients with oromaxillofacial trauma required surgery in the pre-COVID-19 year (PPG) compared to 225 patients during the COVID-19 year (PG). No missing data were found in our series. A statistically significant decrease in the number of fractures in the pandemic group in contrast to the prepandemic group was observed (*p* < 0.001), with an overall decline of 39.36%.

In the PPG, the mean age was 36.01 years (range 3–97), and in the PG, it was 40.17 years (range 5–94) (*p* = 0.56). Most patients were male (74% in the PPG and 77% in the PG), with a male/female ratio of 2.86 and 3.26 in the PPG and PG, respectively. Comparing the sexes, the mean difference was statistically significant with a *p*-value of 0.006. In the PPG, the main etiologies were assaults (41%) and casual falls (27%), while in the PG, there were casual falls (35%) and assaults (35%) (*p* = 0.025). The most frequent anatomical location was the mandible (40% in the PPG and 39% in the PG) (*p* = 0.37).

**Figure 2:**
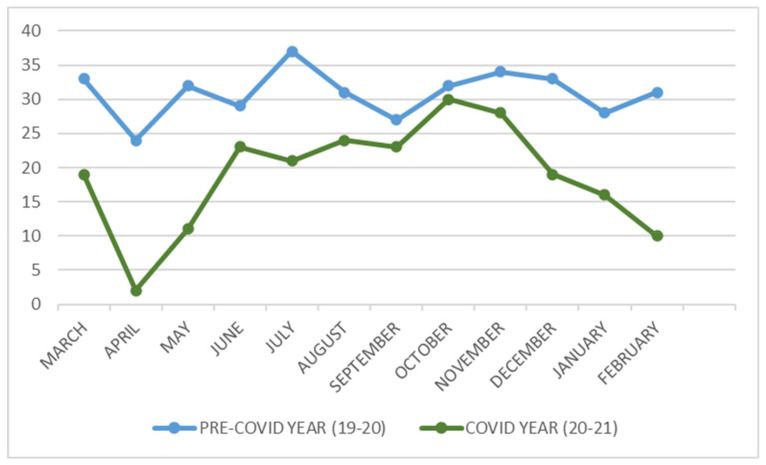
Comparison of facial fractures in each month between the pre-COVID-19 year and the COVID-19 year.

Monthly cases were compared between the two years, and a notable difference was observed, particularly coinciding with the increase in the number of cases and the implementation of restrictive measures by the government. In the PG, the most substantial reduction occurred between April and May 2020, corresponding to the total home lockdown of the population. The month with the largest difference was April (24 vs. 2 fractures). The analysis of incidence according to the confinement periods and mobility restrictions revealed a significant decrease in the volume of fractures during the whole phase of mobility restriction (home lockdown) and different waves (second wave in July). A further striking decrease in fracture cases was observed during the months of December, January, and February, coinciding with the third wave of COVID-19, such as the cancellation of Christmas and New Year’s Eve parties and restrictions on social gatherings (Figure 2).

**Figure 3:**
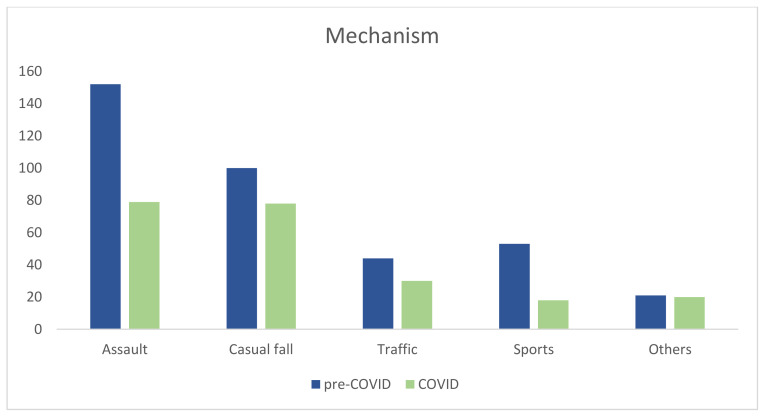
Comparative cause of facial trauma during pre-COVID-19 year and COVID-19 year.

A significant difference in the mechanism of fracture was noticed (*p* = 0.025). We observed a decrease in assaults (41% vs. 35%) and sports-related trauma (14% vs. 8%). There was an increase in casual falls (27% vs. 35%) and precipitation (2% vs. 4%) and a minimal rise in traffic accidents (12% vs. 13%) (Figure 3).

**Figure 4:**
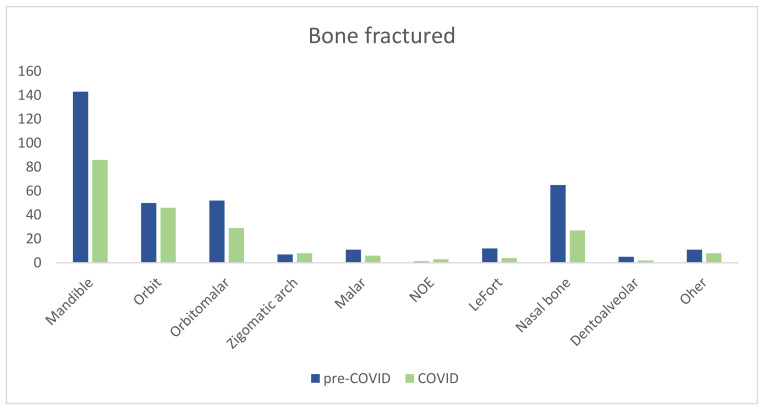
Fracture site during pre-COVID-19 year and COVID-19 year. Abbreviations: NOE (naso-orbit-ethmoidal).

No significance difference was seen regarding the fractured bone (*p* = 0.37). The mandible remained the most commonly fractured bone requiring surgery (40% vs. 39%). We observed an increase in orbit fractures (14% vs. 20%), and the number of nasal fractures requiring surgery was fewer (17% vs. 14%) (Figure 4).

**Figure 5:**
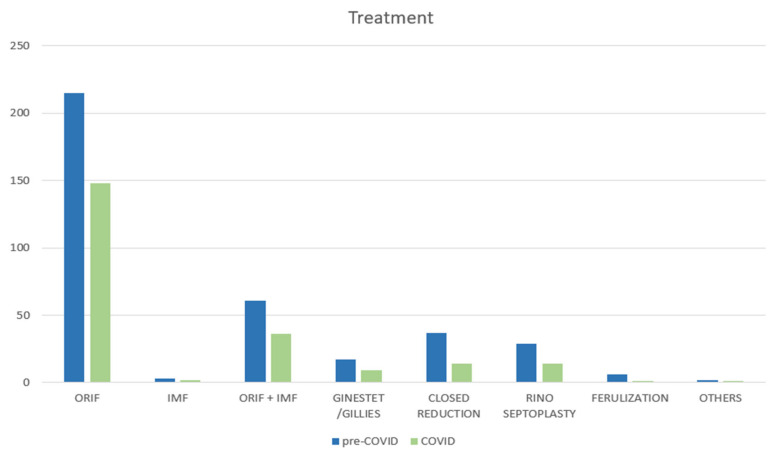
Surgical treatment during pre-COVID-19 year and COVID-19 year. Abbreviations: ORIF, open reduction, internal fixation; IMF, intermaxillary fixation.

No difference was found in the surgical techniques used (*p* = 0.65), with an increase in the open reduction and internal fixation technique (64% vs. 70%) and a decrease in rhinoseptoplasty (9% vs. 7%); all other procedures remained similar when comparing the pre-COVID-19 year to the COVID-19 year (Figure 5).

## 4. Discussion

The transformative effect of COVID-19 on our societal dynamics has been evident, and this study delved into its considerable impact on the incidence of maxillofacial trauma throughout the initial year of the pandemic in Madrid. Our findings disclose a notable 39.36% reduction in total cases (*p* < 0.001), particularly pronounced during the months of April and May 2020, aligning with the period of total home lockdown for the population considered nonessential workers. A graphical representation illustrates this decline in fractures, correlating with the three distinct COVID-19 waves witnessed in the first year.

The literature reports a decrease in non-COVID-19 activity in emergency departments during the pandemic [1,3,4,5]. This disparity could be attributed to changes in outdoor activities, such as a decrease in sports engagement, counterbalanced by an increase in new means of transportation (e.g., electric scooters) and alcohol consumption [6,7,8,9]. Despite challenges in comparability with other studies due to heterogeneity, our results underscore a clear decrease in oromaxillofacial trauma cases, representing the only multicenter epidemiological study in Spain objectively evaluating the incidence variation during various phases of population mobility restrictions. This comparative study considered a full year of pandemic measures and evaluated the association of clinical and therapeutic variables.

The primary impact of pandemic measures was the reduction in surgical oromaxillofacial trauma in Madrid’s pandemic group compared to the pre-pandemic group. Comparable findings were reported by De Boutray et al. [65.5% decline in the first 2 months of the first wave] [2], Fama et al. [55.86% reduction in the first 6 months] [10], and Saponaro et al. [77.5% reduction in the first 2 months] [11]. Patient characteristics and fracture patterns also underwent substantial changes, with the mean age of the pandemic group being higher, aligning with the existing literature [3,11,12].

In developed countries, traffic accidents, assaults, and accidental falls are major causes of facial trauma [7]. The strict lockdown imposed by the Spanish government in March 2020 led to a reduction in interterritorial mobility, sporting events, and social gatherings, impacting the epidemiology of facial trauma. A decrease in assaults and sports-related trauma was observed, consistent with other studies [6,7,10,13,14], accompanied by an increase in casual falls, emerging as the predominant cause during the COVID-19 period, and, unexpectedly, an uptick in traffic accidents. This surge in traffic accidents may be attributed to a preference for private transport postlockdown, avoiding potential infection risks associated with public transport. Increased precipitation during the COVID-19 year, mirroring findings by De Boutray et al. [2], could be linked to heightened psychological distress related to COVID-19 and lockdown [9].

The COVID-19 pandemic has significantly changed the lifestyles of people around the world, impacting alcohol consumption and transportation means. Consequently, policies and safety measures should be devised to address the changes in these factors and mitigate the risk of facial fractures.

Despite changes in epidemiology, the anatomical region affected and the preferred treatment remained consistent. The mandible continued to be the most fractured bone requiring surgery, with open reduction and internal fixation being the favored treatment during the study period.

This multicenter study collected data from most patients treated for oromaxillofacial trauma in Madrid, a region that had a high incidence of COVID-19 during the year of the pandemic and compared the results with those of the previous year. This deliberate timeframe was chosen because, despite experiencing notable changes during the initial period encompassing the first wave of COVID-19, societal norms in terms of daily living and social interactions have not yet fully stabilized. Over this interval, there was a gradual easing of restrictions, marked by the reopening of hospitality establishments, resumption of nightlife activities, and the relaxation of mask mandates in enclosed spaces. Additionally, the emergence of different virus variants led to fluctuating waves, resulting in varying incidence rates of COVID-19-affected patients, culminating in the current seventh wave linked to variants BA4 and BA5 associated with Omicron, contributing to a surge in infections and hospitalizations.

Several studies have focused on shorter durations, yielding comparable outcomes, often concentrating on the initial months or weeks of the pandemic. For instance, Gabriele et al. conducted a 12-week study in Italy, comparing it to the same duration in the four preceding years, revealing a reduction in total cases during the lockdown period [8]. Saponaro et al. observed a reduction in cases and a shift in the causes of facial trauma during the first two months of the pandemic in a tertiary hospital in Rome, compared to the previous year [11]. Nhongo et al. documented a prevalence decrease during two lockdown periods in Melbourne, noting an increase in facial fractures among women due to casual falls and domestic violence, alongside a decline in those resulting from sports, assault, and alcohol-related incidents [15]. Salzano et al. reported a decrease in facial trauma prevalence across six tertiary hospitals in Italy during the initial four months of the pandemic, which was coupled with an increase in the mean age, a decrease in foreign patients, and a reduction in cases related to assault and sports [16]. Philip et al. conducted a one-year study in a single-center in India, describing an increase in casual falls and accidents involving heavy vehicles, a decrease in assaults, and an increase in nose, zygomatico–maxillary complex, and frontal bone fractures [17]. In contrast, Haapanen et al. reported different results, observing no change in cases, although acknowledging modifications in epidemiology during the initial months of COVID-19, with a decrease in assaults but not in alcohol-related trauma [18]. During COVID-19, there was a higher rate of pediatric injuries compared to prior to COVID-19 [19], and the incidence of domestic oral and maxillofacial pediatric injuries increased despite the considered home safety. We did not find this increase in cases in our sample as they are general hospitals, not pediatric hospitals, so, overall, childhood oromaxillofacial fractures had little impact on the overall case mix. In a study carried out in Pakistan, zygomatic bone fracture was most common (53.8%), followed by mandible fracture (31.1%), and only 26% patients were managed under general anesthesia [20]; this difference may be due to difficult access to the operating room in developing patients. In this article from an Iranian group [21], the results are similar to those obtained in our study and differ from the results of the Pakistan study, although the healthcare in both is characteristic of developing countries. In the Iranian group, after social distancing restrictions, there was a significant drop in the number of subjects attending due to motorcycle collisions and road traffic accidents, whereas the number of fractures caused by assaults and domestic violence significantly increased (*p* < 0.001 for each). Marchant et al. [22] found that while the severity of maxillofacial fractures decreased postpandemic and the overall incidence remained the same, Hispanic and firearm-caused maxillofacial fractures increased. This decrease in the severity of trauma has been validated in multiple studies [23,24,25,26,27,28,29,30,31,32], with differences found in published studies on the increase or decrease in emergency department attendance by age group [33,34,35,36]. A possible effect during the most severe moments of the pandemic was the lower willingness to seek medical attention in subacute maxillofacial processes (such as precancerous lesions, cancer, or infections) and the prognostic implication that this implied [30,37]. The availability of adequate protective material and understanding the route of contagion of COVID-19 have made it possible to treat fractures in a similar way in the pandemic and prepandemic periods, with various limitations by country in the most severe peaks of the pandemic [38,39,40,41,42]. In the postpandemic period, various studies have found that health professionals reduced their protective measures [38] due to burnout or psychological reasons, among others. The most recently published articles that studied the influence of COVID-19 on the epidemiology of maxillofacial fractures concluded that the COVID-19 pandemic has produced a change in the epidemiology of oromaxillofacial fractures [43,44,45,46,47,48,49], but the differences observed are specific in each study because the etiology is very diverse and characteristic of the different countries and regions studied. New etiologies have been described, such as the use of electric scooters [50,51], which are producing changes in modes of transportation within cities and even regulatory changes promoting the use of protection measures [52]. It is therefore necessary to publish the findings of studies in different territories because they cannot be extrapolated due to the epidemiological variability and the difference in health resources for treatment [53,54,55,56,57].

Despite the strengths of this study, such as its multicenter design and a substantial number of patients observed over an extended period, several limitations need consideration. The retrospective nature of this study is a primary limitation; however, we contend that our dataset is robust, accurate, and comprehensive, given that all hospital records were electronically documented. Another significant limitation is the inclusion criterion, restricting this study to patients requiring surgical treatment for maxillofacial trauma. This exclusion overlooked cases of minor facial trauma not necessitating surgery, which may have led to a decrease in referrals to tertiary hospitals during the COVID-19 pandemic. While the participating hospitals represented the largest volume of surgically treated oromaxillofacial trauma cases in Spain, the multicenter nature of this study may not have fully encapsulated the nationwide trend. Furthermore, the comparison with the same period from the preceding year was intentional in constructing the control cohort; however, it remains uncertain whether a different duration or approach would yield similar results. Ongoing efforts include the evaluation of oromaxillofacial fractures occurring in the year following the one considered in this study to assess the evolution over more extended periods. Future research should consider prospective multicenter studies to monitor orofacial trauma cases, facilitating efficient resource allocation for prevention and treatment. These studies would also aid in developing hypotheses regarding how the COVID-19 pandemic has impacted this specific pathology, contributing to a more comprehensive understanding of the implications of the pandemic on oromaxillofacial surgical trauma.

The COVID-19 pandemic has decreased the number of surgically treated maxillofacial fractures and changed the epidemiology and etiology of facial trauma. Some measures and interventions to improve the treatment of patients with oral and maxillofacial traumas in the context of potential future pandemics of infectious diseases would be implementing strict infection control protocols to minimize the risk of transmission of infectious diseases during treatment procedures [56]; enhancing personal protective equipment (PPE) guidelines for healthcare professionals to ensure their safety and prevent the spread of infections; developing telemedicine platforms and virtual consultations to reduce the need for in-person visits [57]; strengthening collaboration between oral and maxillofacial surgeons, infectious disease specialists, and public health authorities to develop comprehensive guidelines and protocols; conducting research to identify novel treatment modalities and technologies that can improve the outcomes of patients with oral and maxillofacial traumas; and planning medical and human resources for efficient healthcare during new outbreaks that may occur.

## 5. Conclusions

This study marks the first multicenter investigation aiming to assess the effects of the COVID-19 pandemic on oromaxillofacial trauma in Spain. It involved a comparative analysis of surgical cases treated in the most affected city in the country (Madrid) during the one-year period from the onset of strict lockdown with the corresponding period in the preceding year. Considering the limitations inherent in this retrospective study, we found that the measures implemented to control the transmission of the COVID-19 pandemic have significantly influenced the epidemiology of maxillofacial fractures with a reduction in case volume and alterations in the pattern and characteristics of affected patients.

In conclusion, the COVID-19 pandemic has decreased the number of surgically treated maxillofacial fractures and changed the epidemiology and etiology of facial trauma. Focusing on these changes, it is necessary to develop preventive measures to reduce orofacial trauma, improve safety to prevent the spread of infections, and plan medical and human resources for efficient healthcare in potential new infectious pandemics.

## Figures and Tables

**Table 1:** Summary of patient characteristics, fracture mechanism, bone fractured, and treatment given. Statistical significance, * *p*-value < 0.05. Abbreviations: NOE, naso-orbit-ethmoidal; ORIF, open reduction and internal fixation; IMF, intermaxillary fixation.

	pre-COVID-19 2019–2020 (*n 371*)	COVID-19 2020–2021 (*n 226*)	*p* Value
**Age (years)**			
Range (median)	3–97 (32)	5–94 (37)	
Mean ± SD	36.00 ± 15.58	40.17 ± 18.06	*0.56*
**Sex**			***0.006*** *
Male	275 (74%)	173 (77%)	
Female	96 (26%)	53 (23%)	
**Mechanism**			***0.025*** *
Assault (%)	152 (41%)	80 (35%)	
Casual fall (%)	100 (27%)	78 (35%)	
Traffic (%)	45 (12%)	30 (13%)	
Sports (%)	53 (14%)	18 (8%)	
Precipitation (%)	7 (2%)	11 (5%)	
Other (%)	14 (4%)	9 (4%)	
**Bone fractured**			*0.37*
Mandible (%)	147 (40%)	88 (39%)	
Orbit (%)	51 (14%)	46 (20%)	
Orbitozygomatic (%)	61 (16%)	35 (16%)	
Zygomatic arch (%)	14 (4%)	9 (4%)	
NOE (%)	2 (0%)	2 (1%)	
LeFort (%)	13 (3%)	4 (2%)	
Nasal (%)	64 (17%)	27 (12%)	
Dentoalveolar (%)	6 (2%)	4 (2%)	
Panfacial (%)	10 (3%)	10 (4%)	
Other (%)	3 (1%)	1 (0%)	
**Treatment**			*0.65*
ORIF (%)	215 (64%)	148 (70%)	
IMF (%)	3 (1%)	2 (1%)	
ORIF + IMF (%)	62 (18%)	36 (17%)	
Ginestet/Gillies (%)	17 (5%)	9 (4%)	
Closed reduction (%)	29 (9%)	14 (7%)	
Rhinoseptoplasty (%)	6 (2%)	2 (1%)	
Splinting (%)	2 (1%)	1 (0%)	
Other (%)	0 (0%)	0 (0%)	

## Data Availability

The data that support the findings of this study are available from the corresponding author upon reasonable request.
